# Recent Advances in Electrical Impedance Sensing Technology for Single-Cell Analysis

**DOI:** 10.3390/bios11110470

**Published:** 2021-11-22

**Authors:** Zhao Zhang, Xiaowen Huang, Ke Liu, Tiancong Lan, Zixin Wang, Zhen Zhu

**Affiliations:** 1 Key Laboratory of MEMS of Ministry of Education, Southeast University, Sipailou 2, Nanjing 210018, China; zhangzhao98@seu.edu.cn (Z.Z.); liuke@seu.edu.cn (K.L.); lantiancong@seu.edu.cn (T.L.); 2 The First Affiliated Hospital of Nanjing Medical University (Jiangsu Province Hospital), Department of Orthopedics, Nanjing 210029, China; jiangsuaaa@aliyun.com; 3 School of Electronics and Information Technology, Sun Yat-Sen University, Xingang Xi Road 135, Guangzhou 510275, China; wangzix@mail.sysu.edu.cn

**Keywords:** electrical impedance spectroscopy, impedance flow cytometry, single cell analysis, microfluidics

## Abstract

Cellular heterogeneity is of significance in cell-based assays for life science, biomedicine and clinical diagnostics. Electrical impedance sensing technology has become a powerful tool, allowing for rapid, non-invasive, and label-free acquisition of electrical parameters of single cells. These electrical parameters, i.e., equivalent cell resistance, membrane capacitance and cytoplasm conductivity, are closely related to cellular biophysical properties and dynamic activities, such as size, morphology, membrane intactness, growth state, and proliferation. This review summarizes basic principles, analytical models and design concepts of single-cell impedance sensing devices, including impedance flow cytometry (IFC) to detect flow-through single cells and electrical impedance spectroscopy (EIS) to monitor immobilized single cells. Then, recent advances of both electrical impedance sensing systems applied in cell recognition, cell counting, viability detection, phenotypic assay, cell screening, and other cell detection are presented. Finally, prospects of impedance sensing technology in single-cell analysis are discussed.

## 1. Introduction

Cellular biophysical properties provide signals for abnormalities in tissues and organs [1,2]. Due to the heterogeneity presented in any isogeneic cell population, conventional population-averaged approaches neglect differences among individuals in gene expression and cell processes, leading to the loss of significant information [3,4]. Studies of cellular heterogeneity allow for exploring the cause, diagnosis and targeted therapy of diseases and the discovery of drugs [5,6].

Microfluidics, which provides manipulation and analytical methods at single-cell level, has emerged as a powerful tool for single-cell analysis [7,8]. Microfluidic devices have the advantages of miniaturization, low cost, comparable geometric dimension to cell sizes and flexible structural design [9,10,11]. Various single-cell manipulation strategies have been developed and introduced in microfluidic devices for cell-based studies [12]. These strategies to manipulate cells could be active, such as dielectrophoresis [13], acoustophoresis [14] and optical tweezers [15], or passive, such as microwells [16], hydrodynamic traps [17] and inertial focusing in curved channels [18]. To characterize the diverse biophysical properties of single cells, analytical methods integrated with microfluidic devices have been widely expanded, such as spectroscopy [19], fluorometry [20], mass spectroscopy [21] and electrochemical probes [22]. To study cellular heterogeneity, optical characterization methods, such as optical flow cytometry and laser confocal microscopy, are most widely used to acquire biological information in single-cell resolution [20]. However, these methods require fluorescent labels in cells to characterize cell and subcellular structures.

Electrical impedance properties of single cells could be used as biophysical markers that provide important information to uncover the complex physiological states of cells [23,24,25]. Biosensors based on single-cell electrical impedance measurements have the advantage of probing multiple biological parameters without fluorescent labeling. The electrical impedance at different frequency ranges refers multiple properties of cells: size information at a low frequency (from 100 kHz to 1 MHz), membrane capacitance at a higher frequency (about a few MHz), and intracellular organelles, such as the conductivity of cytoplasm, at even higher frequencies [26,27]. In addition, the non-invasive and label-free impedance sensing techniques are easy to be integrated into microfluidic devices for quantitative and real-time detection of single cells. Due to the advantages mentioned above, electrical impedance integrated microfluidic devices have been widely utilized for cell-based assays in single-cell resolution.

This review first presents a brief overview of basic principles, analytical models and design concepts of electrical impedance sensing devices for single-cell analysis. Next, applications of two essential microfluidic systems for single-cell impedance measurement are focused: impedance flow cytometry for mobile cell detection, such as cell counting, identification, and classification, and electrical impedance spectroscopy for immobilized cell monitoring, such as cell differentiation, division, and proliferation. In the end, advances and prospects on electrical impedance sensing technology for single-cell analysis are discussed.

## 2. Theory and Modeling

In electrical impedance sensing, a frequency-dependent excitation signal in the form of an alternating current (AC) voltage $\tilde{U}\left(j\omega \right)$ is applied to a pair of electrodes and the response current $\tilde{I}\left(j\omega \right)$ is measured. Impedance of the measured object is defined as the ratio between excitation voltage and response current:

(1) $$\tilde{Z}\left(j\omega \right)=\frac{\tilde{U}\left(j\omega \right)}{\tilde{I}\left(j\omega \right)}=Z_{RE}+jZ_{IM}$$

where $\tilde{Z}\left(j\omega \right)$ is the complex impedance. *Z_RE_* and *Z_IM_* are the real and imaginary part of the complex impedance, respectively. *j*^2^ is −1 and *ω* is the angular frequency (*ω* = 2*πf*). The absolute value and phase shift of the complex impedance are given by:

(2) $$\left|\tilde{Z}\right|=\sqrt{{\left(Z_{RE}\right)}^{2}+{\left(Z_{IM}\right)}^{2}}$$

(3) $$\theta =\arctan\left(\frac{Z_{IM}}{Z_{RE}}\right)$$

The common theory and model of cell impedance sensing is the electrical model of a spherical cell in an aqueous medium. The complex impedance of cell-medium system is given by:

(4) $${\tilde{Z}}_{mix}=\frac{1}{j\omega {\tilde{C}}_{mix}}$$

where ${\tilde{C}}_{mix}$ is the complex capacitance of the system and is determined by ${\tilde{\varepsilon }}_{mix}$:

(5) $${\tilde{C}}_{mix}={\tilde{\varepsilon }}_{mix}\frac{S}{4\pi kd}$$

The well-known Maxwell’s mixture theory (MMT) [26,28] is widely used to derive the complex permittivity of mixture of cell and medium as:

(6) $${\tilde{\varepsilon }}_{mix}={\tilde{\varepsilon }}_{m}\frac{1+2\varphi {\tilde{f}}_{CM}}{1-\varphi {\tilde{f}}_{CM}}$$

In this equation, ${\tilde{\varepsilon }}_{mix}$, ${\tilde{\varepsilon }}_{m}$ and ${\tilde{\varepsilon }}_{c}$ represent the complex permittivity of the medium-cell mixture, suspending medium and the cell, respectively. *φ* represents the volume fraction of the cell to the suspending medium and ${\tilde{f}}_{CM}$ represents the Clausius-Mossotti factor:

(7) $${\tilde{f}}_{CM}=\frac{{\tilde{\varepsilon }}_{c}-{\tilde{\varepsilon }}_{m}}{{\tilde{\varepsilon }}_{c}+2{\tilde{\varepsilon }}_{m}}$$

Because MMT only works well in a uniform field with low volume fractions, the volume fraction should be replaced with a corrected value in the cases of high volume fraction (usually >40%) and non-uniform field [27]. Besides, the derivation of MMT is only applicable under homogenous external electric fields and depends on configuration mode of electrodes in specific devices. Considering the geometric parameters of electrode configurations, the impedance of a mixture system can be described as:

(8) $${\tilde{Z}}_{mix}=\frac{1}{j\omega {\tilde{\varepsilon }}_{mix}G_{f}}$$

where *G_f_* is a geometrical constant. Calculation methods of *G_f_* under typical electrode configurations were proposed in previous literatures [28,29,30].

**Figure 1:**
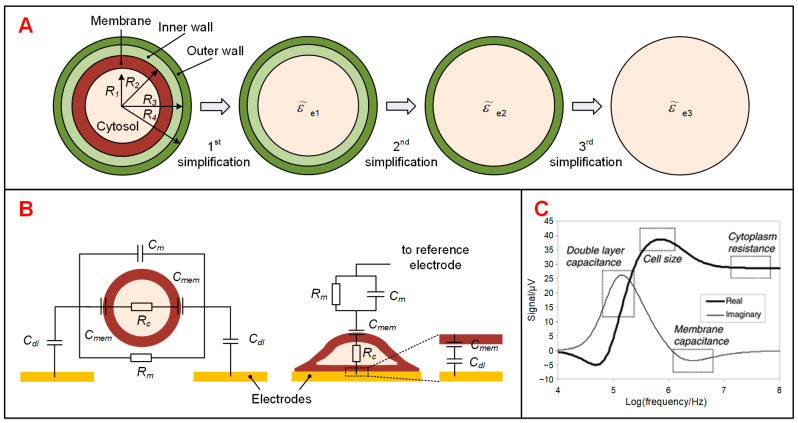
Electrical model and equivalent circuit models (ECMs) of a single cell. (**A**) Multi-shell model of a single cell simplified into a homogeneous sphere based on MMT. (**B**) ECMs of a cell suspended between a pair of sensing electrodes and a cell adhered on a sensing electrode. (**C**) Simulation results of cell impedance sensing using an ECM model, which presented various frequency domains corresponding to different biophysical parameters. Reproduced from [32] with the permission from Royal Society of Chemistry.

The simplest electrical model of a biological cell is the “single-shell model”, which consists of an insulating shell (i.e., plasma membrane) and a conducting sphere (i.e., cytosol, assumed to be homogeneous) [23,27,28]. In addition, plant cells, Gram-negative bacteria like *Escherichia coli* (*E. coli*), and fungi like *Saccharomyces cerevisiae* (*S. cerevisiae*) have outer cell walls. The electrical model of these cells could be represented by “multi-shell model” [31]. Based on MMT, these electrical models could be simplified step by step until it becomes a homogeneous sphere according to following equation:

(9) $${\tilde{\varepsilon }}_{ei}={\tilde{\varepsilon }}_{i+1}\frac{{\left(\frac{R_{i+1}}{R_{i}}\right)}^{3}+2\times \frac{{\tilde{\varepsilon }}_{i}-{\tilde{\varepsilon }}_{i+1}}{{\tilde{\varepsilon }}_{i}+2{\tilde{\varepsilon }}_{i+1}}}{{\left(\frac{R_{i+1}}{R_{i}}\right)}^{3}-\frac{{\tilde{\varepsilon }}_{i}-{\tilde{\varepsilon }}_{i+1}}{{\tilde{\varepsilon }}_{i}+2{\tilde{\varepsilon }}_{i+1}}}$$

where *R_i_* (*i* = 1, 2, 3, 4) and ${\tilde{\varepsilon }}_{i}$ (*i* = 1, 2, 3, 4) are the radius and the complex permittivity of each shell. ${\tilde{\varepsilon }}_{ei}$ (*i* = 1, 2, 3) stands for the complex permittivity of the homogeneous sphere after *i*-th simplification (Figure 1A). After the third simplification, the multi-shell model becomes homogeneous and its complex permittivity (${\tilde{\varepsilon }}_{e3}$) could be substituted into Equation (7) as the complex permittivity of the cell (${\tilde{\varepsilon }}_{c}$).

Typically, a single cell in liquid culture is in either of two conditions: suspended in medium or adhered to the substrate/electrode. Cells in a flow system and cells captured in a fixed position are classified as suspended cells. The ECMs of impedance sensing system with suspended and adherent cells have been provided for theoretically analysis (Figure 1B) [24,26]. In ECMs, cell and medium are simply equivalent to resistors and capacitors in series and parallel according to their electrical parameters. Besides, the electrical double layers (EDLs), formed by the contract between metal electrodes and electrolyte solutions, are modelled as capacitors (*C_dl_*). Ignoring the double layer, the impedance of a mixture system can be described as [24]:

(10) $${\tilde{Z}}_{mix}=\frac{R_{m}\left(1+j\omega R_{c}C_{mem}\right)}{j\omega R_{m}C_{mem}+\left(1+j\omega R_{c}C_{mem}\right)\left(1+j\omega R_{m}C_{m}\right)}$$

where *R_m_* and *R_c_* are the resistance of medium and cytosol, respectively. *C_m_*, *C_mem_* are the capacitance of medium and cell membrane, respectively. Optimizing the dielectric properties of suspension medium can improve the dominance of cell impedance in complex impedance of the mixture. The PBS solution, which has the best conductivity while maintaining the activity of biological cells, is therefore widely used in cell impedance sensing applications.

The frequency range of cell impedance spectra is from 1 Hz to 10 GHz and could be distinguished into three distinct dispersions (or relaxations), which are the well-known α, β and γ dispersions [28]. The α-dispersion, occurs below several kHz, is attributed to the polarization of cell membrane. However, it is difficult to measure due to the domination of impedance by EDLs at low frequencies. At higher frequencies (more than few tens of kHz), the β-dispersion exhibits characteristic sub-dispersions dominated by double layer capacitance, cell size, cell membrane and cytoplasm. Therefore, it is the most widely applied in the electrical impedance sensing of biological samples. The frequency ranges corresponding each sub-dispersion has been given by simulation result using ECMs (Figure 1C) [26]. In GHz range, the γ-dispersion arises from reorientation of water molecules.

## 3. Device Designs for Sensing Single-Cell Impedance

In this section, common designs of single-cell impedance sensing devices are reviewed first, including impedance flow cytometry to sense flowing cells and electrical impedance spectroscopy to sense immobilized cells. Then, instruments and portable platforms to implement impedance sensing are summarized. In the end, complementary metal-oxide-semiconductor (CMOS) based electrical impedance devices are described.

### 3.1. Impedance Flow Cytometry (IFC)

Impedance flow cytometry is a high-throughput methedology for single-cell analysis, analogous to micro Coulter particle counter (μCPC) [32]. Compared with the μCPC, IFC excels in miniaturization, less requirement for peripherals, and flexible integration of interrogation units [33]. IFC measures the variation of response current caused by single cells passing over patterned electrodes in a microfluidic channel. The sensitivity of IFC devices is mainly dependent on the distribution of AC electric field in the channel. Therefore, electrode configurations, namely the geometric setting of electrode pairs, must be considered in particular [34]. Besides, the consistency of detection results can be enhanced by utilizing particle positioning methods to ensure that suspension samples roughly pass through the same cross-sectional position of the sensing region [35,36]. Following subsections will describe several designs of IFC devices, as well as exemplify optimized systems reported recently.

#### 3.1.1. Electrode Configurations

**Figure 2:**
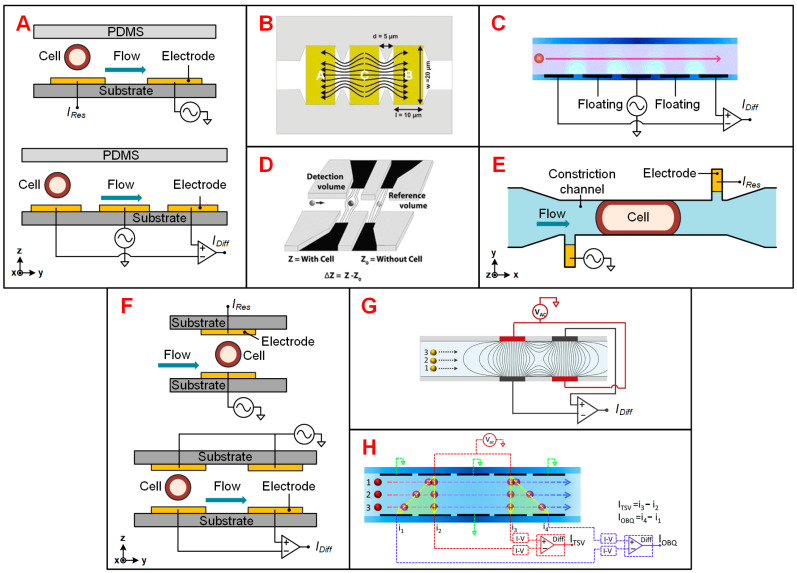
Different designs of IFC devices. (**A**) Schematics of absolute and differential measurement schemes of coplanar electrode configuration. *I_Res_* refers to response current and *I_Diff_* refers to differential current. (**B**) Coplanar electrodes with larger electrode area exposed to the medium. Reproduced from [39] with the permission from MDPI. (**C**) Coplanar electrodes with two extra floating electrodes. Reproduced from [41] with the permission from Royal Society of Chemistry. (**D**) Liquid electrodes. Reproduced from [42] with the permission from Royal Society of Chemistry. (**E**) Asymmetrical liquid electrodes with the constriction channel for cell flowing [43]. (**F**) Schematics of absolute and differential measurement schemes of facing electrode configuration. (**G**) Facing electrodes with asymmetric wiring scheme. Reproduced from [44] with the permission from Elsevier. (**H**) Five pairs of facing electrodes. Reproduced from [45] with the permission from Royal Society of Chemistry.

**Coplanar electrodes.** In 2001, Gawad et al. proposed the first microfluidic IFC device used for high-throughput single-cell impedance measurement at multiple frequencies [32]. As shown in Figure 2A, the basic detection unit in coplanar electrode configurations consists of two or three electrodes positioned in the bottom of microchannel. The absolute measurement scheme facilitates two electrodes to measure the impedance changes in the detection space of the microfluidic channel, which has been discussed in detail in Section 2. The current pulse caused by the passage of a flowing cell is recorded. This scheme is typically applicable for cell counting which has no requirement of detecting small signal changes [37]. In a differential measurement scheme, which has three electrodes, excitation signal was applied to the intermediate electrodes and the differential current was measured at the electrodes on both sides to provide a higher signal to noise ratio (SNR) [32]. Such a configuration enabled automatically switching between the measurement and reference electrode pairs when a single cell passing through the sensing region. The signal waveform of the differential current was a symmetric bipolar Gaussian shape and gave the information about cell size and electric parameters upon frequencies. Compared to the absolute measurement scheme, differential measurement is widely used since it corrects uneven drift of electrode properties and enables the calculation of the flow rate of cells [38,39,40]. The devices with coplanar electrodes can be easily fabricated with tiny deviations due to the single-step alignment between the channel and metal [32].

However, IFC devices with coplanar electrodes are sensitive to the vertical position of samples because of the non-uniform distribution of electric field. Hence, several optimizations of coplanar electrode configurations were proposed to improve the sensing sensitivity. As shown in Figure 2B, Clausen et al. proposed an optimized chip with larger electrode area exposed to the medium [39]. This design allowed for more current and greater current density between the electrodes, and thus attained an improved SNR. Xie et al. adopted a similar design and their IFC device thus had a higher SNR (23.5–32.6 dB) [46]. Besides, they proposed that this design scheme enabled lower amplitude of excitation signal to reduce the potential damage to cells. Rather than seeking ways to increase current intensity or local electric intensity, De Ninno et al. proposed a five-electrode design combining a conventional chip layout with compensation strategy which enabled accurate size measurement of particles without the need for focusing methods [41]. In this device, one floating electrode was placed between each pair of the detection electrodes to obtain more information with respect to the height of the particles (Figure 2C). As a result, the proposed compensation procedure made the “electric” diameters (the estimated diameter of particle) closer to the actual data. Liquid electrodes proposed by Demierre et al. were designed to address the non-uniform electric field in conventional coplanar electrode configuration (Figure 2D) [42,47]. In these devices, electrodes were located at both ends of short channels perpendicular to the main channel. The electrodes were far enough to generate almost homogeneous electric field over the channel height. However, this design reduces the sensitivity due to the larger detection volume. Besides, the liquid electrode designs are also used for focusing particle stream based on the principle of dielectrophoresis [48,49].

In order to compare the performance of different coplanar electrode designs, Cottet et al. evaluated the vertical and lateral sensitivity of four typical layouts [50]. As a result, the design with a longer constriction channel was considered as the best candidate since it was relatively insensitive to the particle height or longitudinal misalignment in the fabrication process.

**Facing electrodes.** Facing electrode design was firstly proposed by Cheung et al. [51]. In this configuration, the electrodes are positioned at the top and bottom of the channel and thus eliminate the electric field non-uniformity to some extent (Figure 2F). As the electric field is limited to smaller volume, this design has improved sensitivity largely. Absolute measurement schemes are usually used for simple cell counting and therefore require less sensitivity. Thus, absolute measurement schemes are rarely used in practical applications, while differential measurement scheme is widely used in many prototypes [52,53,54]. However, the fabrication process of facing electrode design needs accurate alignment of electrodes patterned at different substrates, leading to higher fabrication difficulty.

In order to obtain impedance information related to the vertical height of the single cells, Caselli et al. proposed an asymmetric wiring scheme (Figure 2G) [44]. This solution can improve the precision level of particle diameter measurement to a certain extent and simply compensate the signal impedance of eccentric particles. Besides, in order to obtain impedance data with high accuracy, Spencer et al. proposed a new design of multiple pairs of facing electrodes with an impedance signal processing algorithm, minimizing the influence of the vertical height of the particle on the impedance signal (Figure 2H) [45].

#### 3.1.2. Particle Positioning

The relative position of particles passing through the detection area between electrode pairs has great effects on the detection stability and repeatability, especially in coplanar electrode design [37,55,56]. Except for reducing the cross-sectional area of the channel, constriction channels and various methods of focusing particles have been proposed in order to obtain higher impedance signal quality.

**Constriction channel.** Due to the lack of close contact between cells and electrodes when cell passing through the detection area, the electric current tends to bypass the cells by flowing through the surrounding medium. In order to solve this problem, Chen et al. applied the constriction channel design in a μCPC device [57]. In this design, cells were pressed and elongated when flowing into the constriction channel. Constriction channel allows for more accurate detection of specific membrane capacitance and cytoplasm conductivity, thus increasing the classification success rate of different cell populations [58]. Hence, it has been used for screening various types of single cells, such as blood cells [59], tumor cells [43,58] and plant cells [60]. Due to the cross-sectional area of the constriction channel must be smaller than the size of the interested cells, this design has higher risk of channel blockage and lower throughput. To this end, Zhao et al. proposed the crossing constriction channel design with bypass outlet, which allowed large particles passing through to address the possible blockage of the constriction channel [58]. Furthermore, in order to improve the accuracy of cell classification, they introduced asymmetrical liquid electrodes to obtain cell diameter (Figure 2E) [43]. In this method, the measured impedance is proportional to the elongated length of the cell, so that the relative volume of the cell can be calculated. The passage time for cells to pass through the constriction channel is related to cellular mechanical properties. In order to obtain the passage time, Han et al. introduced a pairs of coplanar electrodes at the inlet and outlet of the constriction channel [60]. The time point when a cell passes through each sensing unit is recorded, and then the passage time can be calculated according to the time interval for a cell from a sensing unit to another.

**Particle focusing.** Although particle focusing system sometimes increases the device complexity, it is commonly integrated in IFC devices for eliminating the influence of sectional position on impedance signals. Various particle focusing systems based on different technologies have been reported such as hydrodynamic focusing [37,61,62], acoustophoretic focusing [63,64,65], dielectrophoretic focusing [66,67] and inertial focusing [68]. Hydrodynamic focusing enables the sample flow to be coated by sheath flow and focused into a narrow stream either horizontally or vertically. This conventional method minimized the potential for two or more particles to enter the detection region simultaneously and ensures a uniform particles velocity. Acoustophoretic focusing methods are based on either traveling surface acoustic waves (TSAWs) [64] or standing surface acoustic waves (SSAWs) [65] to manipulate particles. The principle of dielectrophoresis (DEP) focusing is that a neutral but polarizable particle is subjected to DEP force in a nonuniform electric field. Since the DEP forces depend on the size and dielectric properties of the particles, it can also be used for single-cell trapping and separating. Inertial focusing, as a passive focusing method, depends on special channel structure and high particle flow velocity.

### 3.2. Electrical Impedance Spectroscopy (EIS) Sensing Devices

EIS sensing is suitable for real-time monitoring and tracking of a limited number of cells simultaneously. For *in-situ* EIS measurements, frequency sweeping takes several seconds every time, which requires the stable capture of single cells as a prerequisite [24]. Therefore, various trapping methods have been proposed for positioning single cells in microfluidic channel. Alternatively, single cells can be directly adhered onto the electrode or substrate for electric cell-substrate impedance sensing. Additional progress has been also made to increase the throughput of EIS devices.

#### 3.2.1. Trapping of Suspended Single Cells

**Figure 3:**
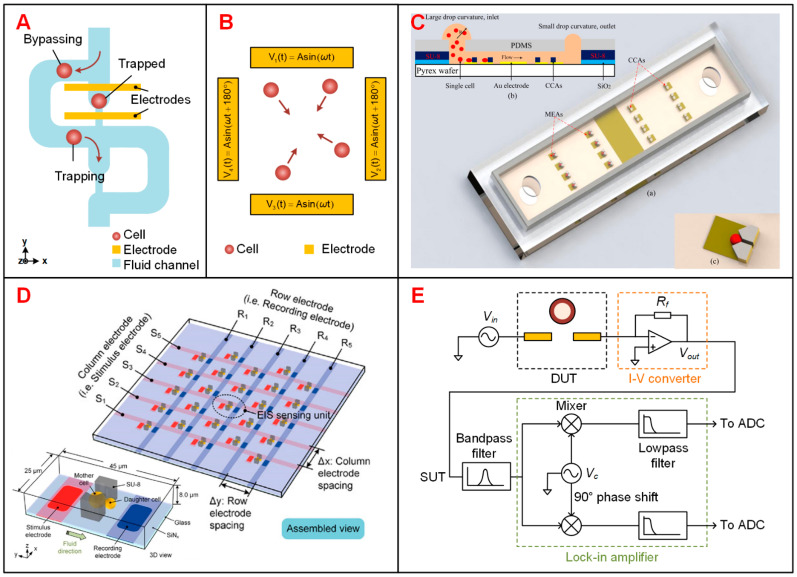
Different designs of EIS devices and schematics of single-cell EIS sensing system with lock-in amplifier. (**A**) Schematics of μ-fluidic traps to immobilize single cells in a cellular EIS sensing device [82]. (**B**) Quadrupole electrode array to gather cells towards the center. The phase difference of applied AC signals between adjacent electrodes is 180° [78]. (**C**) A three-dimensional (3D) single-cell culturing device to detect HeLa cell migration. Reproduced from [85] with the permission from American Chemical Society. (**D**) An addressable microelectrode array to perform single-cell immobilization and localized EIS measurement. Reproduced from [86] with the permission from John Wiley and Sons. (**E**) Schematics of single-cell impedance sensing system with lock-in amplifier.

The methods to trap suspended single cells include hydrodynamic traps [69,70,71,72,73], negative pressure traps [74,75,76], DEP trapping [77,78,79,80] and optical manipulation [81]. Hydrodynamic traps are the most common design to position single cells in microfluidic devices, and usually consist of simple microstructures (such as U-shaped or three-pillar traps [69,71,73]) or special fluid channels (such as μ-fluidic traps [70,72]) to dock numerous single cells synchronously in a short time. Di Carlo et al. captured approximately 100 isolated HeLa cells by a U-shaped trap array and achieved the cell maintenance over 85% after 24 h [69]. Tang et al. applied the μ-fluidic microstructures to achieve a high cell-trapping rate of 95% (Figure 3A) [82]. In negative pressure trapping system, one or more suction channels are connected to the side or bottom of the cell-perfusion channel [74,75,76]. By applying negative pressure to the suction channel, single cells could be trapped in vias or slits which are the connection points of cell-perfusion channel and suction channels. Although such devices are complex in design and fabrication, negative pressure traps allow for selective capture and release of redundant or unwanted cells [76]. Han et al. demonstrated a system with backside suction channels to capture HeLa cells in cavity pores [75]. Since the diameter of the pore is between that of HeLa cells and blood cells, most of the HeLa cells could be separated from the spiked blood samples. DEP trapping could be used to perform accurate and selective capture of specific samples, as the DEP force depends on the dielectric properties of single cells and the frequency of applied electric field [79]. Besides, DEP force was also used to release redundant cells to achieve a uniform single-cell trapping [83]. Electrode configuration schemes for DEP trapping include quadrupole electrode array [77,78], microwells [79,80], and ‘ring-dot’ electrode structures [84]. As a representative, the quadrupole electrode array, proposed by Heida et al., could generate DEP force to repel cells away from electrodes and towards the array center (Figure 3B) [78]. Optical methods were used to manipulate single cells with a high precision. The liquid resin containing target cells could be rapidly polymerized under laser exposure and formed traps of specific shape. As such, Xu et al. performed real-time two-photon-lithography to capture single cells and achieved a high capture efficiency of 100% on a one-bead-to-one-trap basis [81].

#### 3.2.2. Electrical Cell-Substrate Impedance Sensing (ECIS)

ECIS is a well-established technology developed to assess cellular behavior or responses to drug candidates by measuring the impedance of live cells adhered on the electrode surface [87,88]. As cells proliferate and spread on the surface of sensing electrodes, electrical current is interfered immediately and thus resulting in a drastic change in the measured impedance. Besides, ECIS could combine with a variety of single-cell manipulation techniques to perform single-cell impedance measurement [85,89,90,91,92]. Tsai et al. [91] used micro pillars to trap single HeLa cells in a microfluidic system and monitor their adhesion and spreading Zhang et al. applied DEP trapping to HeLa cells and monitored their adhesion, response to drugs, steady growth and differentiation by ECIS [92]. In addition, surface modification could be used to promote cell adhesion on metal electrodes rather than glass or plastic substrate [87,93]. Shah et al. modified the surface of the SU-8 substrate sequentially with mNH_2_ linked PEG (Amino functionalized methoxyl polyethylene glycol) to avoid any nonspecific cell adhesion near the sensing electrode and, thus, eliminate the unnecessary cell crosstalk [93]. Nguyen et al. proposed a microfluidic chip with removable PDMS cover lid which enabled building up a two-dimensional or three-dimensional microenvironment for investigating single cancer cell migration (Figure 3C) [85].

#### 3.2.3. Advanced Design to Increase the Throughput of EIS Devices

Typically, conventional EIS devices can monitor less than 20 cells simultaneously [94,95]. Although simple devices are useful for low-cost assays, they result in tedious repetition of experiments and reduced data reliability in applications, which usually require long-term monitoring of a large population of samples. In these devices, on-chip impedance sensors are connected with respective bond pads for external interconnection [83,85], where the dimension of sensors is limited by the chip margin. To solve this problem, individually addressable microelectrode arrays (MEAs) are proposed to be incorporated into EIS sensing devices [86,96]. In these devices, two sets of *n* microelectrode bars are arranged orthogonally to form a sensing array of *n* × *n* sites with only 2*n* bond pads (Figure 3D) [86]. Each sensing unit at the crossing position can be addressed individually by external multiplexers. Guo et al. reported a microarray chip integrated with two MEAs for cell positioning and impedance monitoring, respectively [96]. Geng et al. proposed the design of a MEA chip to measure the replicative lifespan (RLS) of budding yeasts and assessed the influence of neighbor samples upon the site-specific impedance measurement [86]. Alternatively, CMOS integrated circuit could be utilized to overcome the limitation of the number of EIS sensing units by integrating electrodes and addressing circuit (see Section 3.4).

### 3.3. Instruments and Portable Platforms for Electrical Impedance Sensing Technology

Impedance converter and lock-in amplifier (LIA) are commonly used to measure the impedance of single cells. The impedance converter is an AC self-balancing bridge, which consists of a simple op-amplifier and a feedback resistor (Figure 3E) [97]. In this approach, an AC excitation signal *V_in_* is input to one port of the device under test (DUT), and the feedback resistor *R_f_* shares the same current flowing through the DUT. Ideally, without any phase shift of the op-amplifier, the current flowing through the DUT is proportional to the voltage on *R_f_*. Then the DUT complex impedance can be calculated from the output voltage *V_out_*. LIA, also known as the phase-sensitive detector, is capable of extracting weak signals from noisy background (Figure 3E) [98]. The output voltage *V_out_*, termed as signal under test (SUT), is multiplied by in-phase and quadrature carrier signal *V_c_*, whose frequency is the same as that of *V_in_*. Then, the amplitude and phase of SUT are extracted with correlative demodulation by carrier signal at the same reference frequency. Benchtop instruments, including several versions of LIAs developed by Stanford Research Systems Inc. (Sunnyvale, CA, USA), Zurich Instruments AG (Zurich, Switzerland), NF corp. (Yokohama, Japan), SBT instruments (Copenhagen, Denmark), Sine Scientific Instruments (Guangzhou, China) and Liquid Instruments (Lyneham, Australia), respectively, are usually interconnected directly or through custom printed circuit boards (PCBs) to the microfluidic devices [99,100,101,102,103,104]. Here, PCBs are typically functionalized with control modules such as multiplexers to activate target sensing electrodes on the devices.

Device integration leads to cost saving, while portable system serves for more situations. Motivated by this, researchers began to integrate impedance measuring circuit on custom PCBs. As a representative, the integrated circuit chip AD5933, a low-cost impedance analyzer system, has been introduced in embedded portable systems [105]. Notably, the frequency range of this system is only up to 100 kHz, and its accuracy is lower than that of benchtop instruments. Huang et al. developed a wide-band digital lock-in amplifier (DLIA), which features a low input noise of 4.4 $\mathrm{nV}/\sqrt{\mathrm{Hz}}$, 120 dB dynamic reserve and a phase deviation of less than 0.02° through the whole frequency range up to 65 MHz [106]. This portable EIS system has been demonstrated by impedance measurements of three sets of micro beads with different diameters.

### 3.4. CMOS-Based Impedance Sensing Devices

The miniaturization and portability of single-cell impedance sensing devices can be achieved by harnessing CMOS integrated circuit (IC) technology. CMOS IC chips have been reported as an alternative to measure biological impedance at single-cell level, showing the trend of impedance sensing system in the integration level [107,108]. However, the aluminum (Al) microelectrodes fabricated by standard CMOS processes are not biocompatible due to the biological toxicity and chemical activity of Al. Therefore, the microelectrodes are covered with gold (Au) or platinum (Pt) layer to obtain better biocompatibility and chemical inertness [107]. Since its substrate is replaced by large scale integrated circuits, such devices enable tens of thousands of sensing units featuring individually addressable microelectrodes. Chen et al. developed a high-throughput EIS sensing platform consisting of a 96 × 96 microelectrode array for tumor cell counting and analysis [107]. This CMOS chip was packaged with a PCB that contains multiplexers and the EIS measurement of cells was implemented by a LCR meter. The size of microelectrodes approximates to the scale of target cells for better sensitivity, resulting in a weak current of about 100 pA that is easily submerged in the noise. To overcome this problem, Gamo et al. introduced a current integrator acting as a I-V converter to effectively suppress noise and measure weak current signals [108]. More efforts are required to achieve the complete integration of impedance sensing circuitry on CMOS chip. Visvam et al. reported a high-density CMOS MEA system, including a programmable waveform generator, 59,760 platinum microelectrodes and 32 on-chip lock-in amplifiers for impedance sensing [109]. This improved integration level contributes to superior sensing and actuation capabilities and high signal quality.

## 4. Applications of Single-Cell Impedance Sensing Technology

### 4.1. IFC to Detect Flowing Single Cells

Recent IFC devices applied in single-cell analysis are summarized in Table 1. These applications, discussed in this subsection, are simply classified according to cell species, including blood cells [110,111,112,113,114], tumor cells [43,52,115,116,117,118,119,120,121,122], stem cells [123,124,125,126,127], plant cells [60,128,129,130,131,132] and microbes [53,62,133,134,135,136,137,138,139,140,141]. In terms of blood cells, researchers focused on the identification and counting of normal or diseased blood cells. Studies showed the capability of IFC devices in recognition of different types of dissociated tumor cells (DTCs) [43,118,120,121] or circulating tumor cells (CTCs) [52,115,119,122]. As for stem cells, the main focus is the impedance measurement of their long-term differentiation process. Studies of plant cells include the detection of pollen viability [128,129,130,131,132] and cell screening [60]. Besides, impedance measurements of microbes are further classified and discussed.

#### 4.1.1. Blood Cells

**Figure 4:**
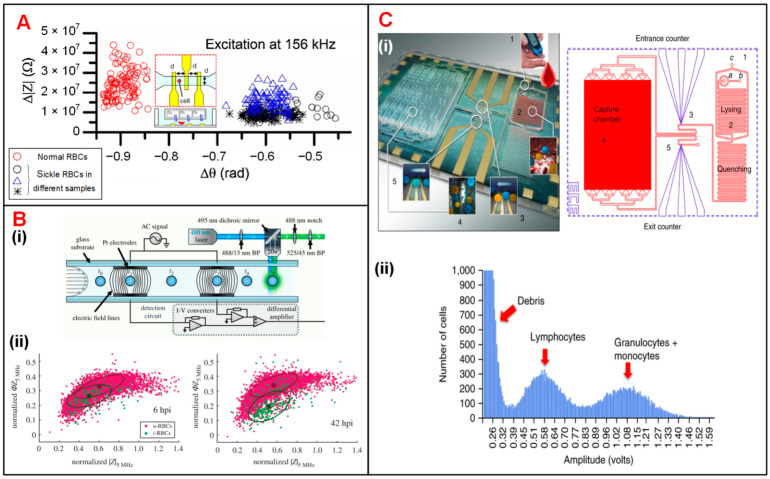
Blood cell analysis using IFC devices. (**A**) Measurement of Δ|Z| vs. Δθ for normal and sickle RBCs at 156 kHz. Reproduced from [110] with the permission from Elsevier. (**B**) (**i**) Schematics of a IFC device integrated fluorescence detection. (**ii**) Measurement of normalized phase (*Φ*Z_5MHz_) vs. amplitude (|Z_5MHz_|) at 6 h and 42 h after RBC infection. u-RBCs and i-RBCs stand for uninfected and infected RBCs. Reproduced from [114] with the permission from Royal Society. (**C**) (**i**) Photograph and layout of the differential immunocapture biochip. (**ii**) Pulse amplitudes of recorded impedance signals showing the size distribution of cells. Lymphocytes and granulocytes + monocytes are two groups of distinct populations of leukocytes. Reproduced from [111] with the permission from Springer Nature.

Sickle cell disease (SCD), which causes sclerosis and membrane distortion in red blood cells (RBCs), brings about variation in cellular electrical properties [142]. Liu et al. combined on-chip oxygen control onto a single IFC chip for sickle cell disease diagnosis and monitoring [110]. They measured the electrical impedance of normal cells and sick cells at three different frequencies under normoxic and hypoxic conditions, respectively. As shown in Figure 4A, normal RBCs and sickle cells were separated clearly according to the measured impedance amplitude and phase value at 156 kHz under the normoxic condition. The results suggested that electrical impedance could serve as a new parameter to diagnose sickle cell disease. Parasite invasion can alter the dielectric properties of RBCs [113,143]. Du et al. demonstrated the discrimination of normal RBCs and *P. falciparum*-infected RBCs through analyzing the changes in the impedance signal amplitude and phase [113]. Honrado et al. developed an IFC device integrated with fluorescence interrogation to detect the dielectric properties of RBCs infected by malaria (Figure 4(Bi)) [114]. As a result, for early-stage infection (6 h), infected cells and normal cells were not distinguishable according to their impedance signals. However, as parasite growth progressed, the membrane capacitance and cytoplasmic conductivity of infected RBCs increased and thus the discrimination between two cell populations gradually became detectable (Figure 4(Bii)).

Holmes et al. discriminated and enumerated CD4 T-cells based on impedance cytometry and immune capture [112,144]. In their study, CD4 T-cells were labeled with small antibody conjugated beads, which changed the electrical properties of target T-cells. Hence, CD4 T-cells could be identified from their corresponding subpopulations based on impedance opacity (|Z_10MHz_|/|Z_503kHz_|). Recently, Hassan et al. reported an impedance biosensor based on differential immunocapture technology to perform cell counting on CD4 and CD8 T-cells with high accuracy (Figure 4(Ci)) [111,145,146]. In this device, antibodies specific to CD4 T-cells were initially adsorbed on a chamber between two conventional IFC modules. As the leukocytes flowed into the chamber, CD4 T-cells were captured and immobilized on the antibodies (Figure 4(Cii)). The cell number of each population can be calculated according to the impedance pulses caused by the passage of cells through the chamber. This protocol can be used to enumerate specific cell types with their corresponding antibodies immobilized in the capture chamber.

#### 4.1.2. Tumor Cells

**Figure 5:**
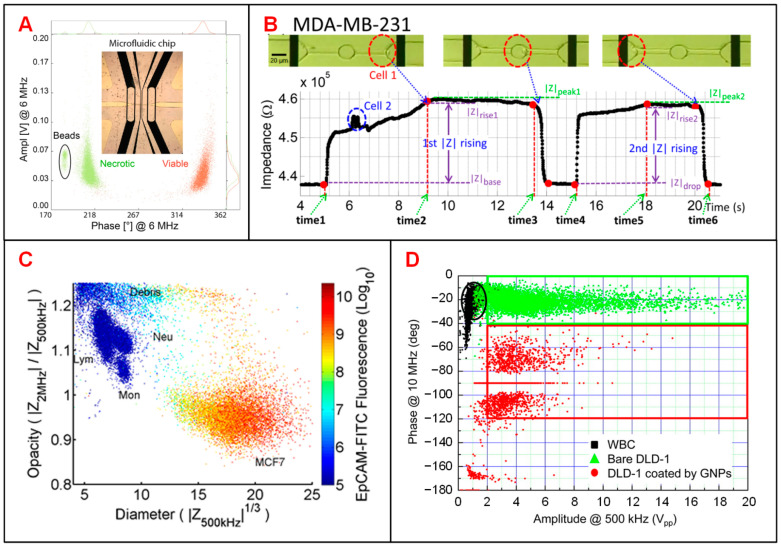
Tumor cell analysis using IFC devices. (**A**) Scatter plot of amplitude and phase values at 6 MHz for necrotic and viable U937 human lymphoma cells and 10-μm beads. Reproduced from [121] with the permission from Springer Nature. (**B**) Recording of the impedance variation when a breast cancer cell (MDA-MB-231) passing through the constriction channel. Reproduced from [119] with the permission from American Institute of Physics. (**C**) Scatter plot of opacity (|Z_2MHz_|/|Z_500kHz_|) and electric diameter (|Z_500kHz_|^1/3^) for MCF-7 cells and other blood cells. Reproduced from [52] with the permission from AIP Publishing. (**D**) Scatter plot of impedance amplitude (|Z_10MHz_|) and phase (*Φ*Z_500kHz_) to classify white blood cells, bare DLD-1 cells and DLD-1 cells coated by GNPs. Reproduced from [116] with the permission from American Chemical Society.

Tumor diagnosis is underpinned by determining which cells are malignant in acquired biopsy, leading to the need to accurately distinguish DTCs from normal cells in tissue [2]. Zhao et al. classified two tumor cell lines (A549 and H1299) based on different cellular membrane capacitance (C_m_) and cytoplasm conductivity (σ_p_) [117]. Desai et al. separated lung cancer DTCs (LC-DTCs) from RBCs, peripheral blood mononucleated cells (PBMCs) and normal lung cells based on impedance amplitude [118]. When LC-DTCs pass through the coplanar electrodes, impedance amplitude signal generates more significant pulse compared to that of normal cells. They also determined five major cancer types (lung, thyroid, breast, ovarian, and kidney cancers) from their corresponding counterpart target cells. Pancreatic ductal adenocarcinoma (PDAC) is an aggressive cancer lacking specific biomarkers. Aimed at this situation, McGrath et al. reported an IFC device to separate single PDAC tumor cells against xenografts [120]. They found that the phase of impedance signal of six PDAC cell types showed some correlations to specific gene expression, especially the *KRAS* mutations that led to higher phase variation. T188 and T738 are primary stage tumors with unknown *KRAS* mutations showing lower impedance phase contrast than other PDAC samples. Zhang et al. developed a microfluidic IFC platform with asymmetrical constriction channel to better detect the dielectric properties and diameters of different types of single tumor cells (Figure 2E) [43]. The classification accuracy between two tumor cell lines, A459 and HEP G2 cells, could be significantly improved with the combination of the individual intrinsic bioelectrical markers of membrane capacitance, cytoplasm conductivity and cell diameter. Besides, Ostermann et al. reported that necrotic and viable U937 human lymphoma cells could be clearly discriminated based on the phase of impedance signals by using a commercial IFC device [121]. Dead and viable cells can be discriminated by impedance signals at high frequency as the imaginary component of cell impedance depends on the membrane integrity of the cell (Figure 5A).

Identification and characterization of CTCs in blood stream is key to monitor the progression of cancer metastasis [122]. Choi et al. proposed a simple DC impedance microcytometer for identifying CTCs according to the cell volume [115]. Ren et al. reported an IFC device featuring parallel cyclic deformability channels and coplanar electrodes, to collect both biomechanical and bioelectrical properties for tumor cell analysis [119]. The deformation and transition time of tumor cells could be obtained from the time points when impedance amplitude changes abruptly (Figure 5B). In clinical application, due to the very small amount of CTCs in a blood sample, it is necessary to pre-enrich CTCs before measurement [52,116]. According to the different membrane capacitance between tumor cells and normal PBMCs, Spencer et al. measured the dielectric properties of MCF-7 cells (a representative of CTCs) at 500 kHz and 2 MHz and distinguished them from leukocytes when mixed in the whole blood [52]. Compared with optical approaches, electrical impedance measurement shows better performance in separating MCF-7 cells from other blood cells (Figure 5C). Besides, Han et al. reported a microfluidic system integrated with both enrichment and impedance detection units to discriminate CTCs [116]. In this study, immunomagnetic nanobeads (MNBs) and highly-conductive graphene nanoplates (GNPs) were bonded to the surface of DLD-1 cells (a representative colorectal cancer cell line). Compared with normal blood cells, the impedance signal of DLD-1 cells coated with GNPs shows a phase shift of 100 degrees for identification (Figure 5D), successfully.

#### 4.1.3. Stem Cells

**Figure 6:**
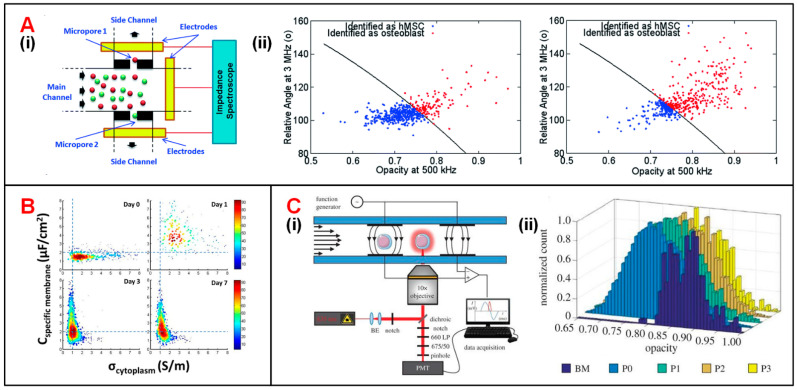
Stem cell analysis using IFC devices. (**A**) (**i**) Schematics of a dual-micropore based IFC device consisting of a main channel and two deputy channels through micropores. (**ii**) Scatter plot of signal phase at 3 MHz vs. opacity (|Z_3MHz_|/|Z_500kHz_|) for hMSCs and osteoblasts at 7 days (on the left) and 14 days (on the right) after post-induction. Reproduced from [125] with the permission from Royal Society of Chemistry. (**B**) C_specific membrane_ and σ_cytoplasm_ variations of rat neural stem cells within the differentiation process of 7 days. Reproduced from [126] with the permission from Public Library of Science. (**C**) (**i**) Schematics of an IFC device integrated fluorescence detection. (**ii**) Changes of impedance signal opacity (|Z_2MHz_|/|Z_500kHz_|) within 56 days SSCs differentiation process. Reproduced from [127] with the permission from Royal Society.

Hildebrandt et al. demonstrated that the osteogenic differentiation process of human mesenchymal stem cells (hMSCs) could be monitored by tracking their impedance variation [123]. Song et al. was the first to propose a dual-micropore microfluidic IFC device to monitor the same differentiation process (Figure 6(Ai)) [124,125]. In this device, when MSCs or osteoblasts passed through micropores, a pulse in impedance amplitude was recorded to determine the proportion of differentiated cells at each stage [125]. Moreover, a support vector machine (SVM) algorithm was employed in data analysis to reach a classification accuracy of 87%. It is notable that the training data set of SVM included a total number of 1028 impedance signals combining both relative phase at 3 MHz and impedance opacity (|Z_2MHz_|/|Z_500kHz_|). The optimal SVM-based model was also used to characterize the differentiation process (from MSCs into osteoblasts), in which the proportion of osteoblasts was increasing while that of MSCs was decreasing (Figure 6(Aii)). Zhao et al. reported that the intercellular electrical markers, such as specific membrane capacitance (C_specific membrane_) and cytoplasm conductivity (σ_cytoplasm_) of neural stem cells could be used to evaluate their differentiation processes [126]. During different stages of differentiation, the distribution difference of C_specific membrane_ differs a lot from that of σ_cytoplasm_ (Figure 6B). Besides, Xavier et al. developed an IFC device equipped with two pairs of facing electrodes combined with confocal microscopic monitoring of the osteogenic differentiation of skeletal stem cells (SSCs) (Figure 6(Ci)) [127]. The sample of human bone marrow mononuclear cells (hBMMNCs) extracted from human bone marrow (BM) were cultured in vitro and was injected into a microfluidic chip for impedance detection every two weeks. Changes in the opacity (|Z_2MHz_|/|Z_500kHz_|) of impedance data could characterize the osteoblast differentiation process of SSCs. (Figure 6(Cii)). In the first two weeks (from BM to P0), the decreased opacity corresponded to the increased cell size and membrane capacitance during SSCs osteogenic differentiation. After P0, there was no significant change of opacity indicating the completion of osteogenic differentiation.

#### 4.1.4. Plant Cells

Qualifying pollen or spores, especially their viability and germination capacity, is important for industrial production and plant breeding [128]. Heidmann et al. measured the viability of pollen samples by using a commercial impedance device [128,129]. In one of these studies, pollen samples were measured before and after heat treatment [128]. As a result, larger phase of the impedance signal corresponding to viable samples was no longer presented after heat-inactivation, which suggested that heat treatment inactivated the pollen samples and destroyed the integrity of cell membrane. Furthermore, Heidmann et al. predicted the germination rate of tomato pollen population by measuring the amount of viable and dead pollens [129]. Impe et al. [130] and Ascari et al. [131] assessed pollen viability of hazelnut and wheat, and further identified various factors (sugar, H_3_BO_3_, CaCl_2_·2H_2_O/Ca(NO_3_)_2_·2H_2_O concentration and pH) affecting pollen viability with the same commercial device. Canonge et al. utilized the IFC device to track and characterize the developmental process of wheat (*Triticum aestivum* L.) genotype Pavon microspores in gametogenesis and anrogenesis [132]. According to this study, throughout all sporophytic developmental stages, some of the viable microspores showed a continuous increase in both impedance amplitude and phase. As a result, electrical impedance could serve as a fast and reliable reactivity marker for tracking wheat microspores in androgenesis.

The biological and physiological properties of cell wall unique to plant cells offer the potential to increase phenotyping resolution and identify nonanatomic markers [147]. Han et al. developed an IFC device to characterize the biophysical properties of two model plant species, herbaceous *Arabidopsis thaliana* and woody *Populus trichocarpa* [60]. In the regeneration process of primary cell wall (PCW), plant cells are gradually covered by the fibrillary network, which becomes thick and interlaced, resulting in the decrease of capacitance of cell membrane and PCW [148]. Thus, the researchers found that the *Arabidopsis* cells with regenerated PCW were less deformable and electrically conductive than that without PCW.

#### 4.1.5. Microbes

IFC devices have been utilized extensively in detection, separation and viability analysis of unicellular microbes, including bacteria [53,62,133,134,135,140,141], protozoa [136,149] and fungi [137,138,139].

**Figure 7:**
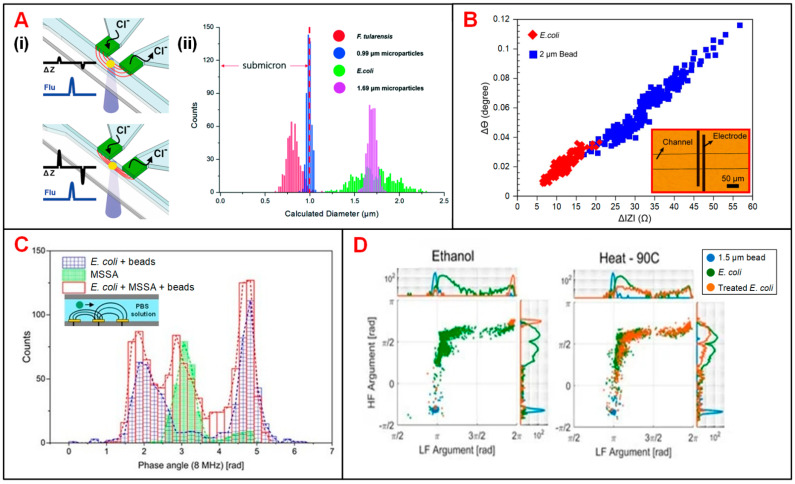
Bacteria analysis using IFC devices. (**A**) (**i**) Schematics of an IFC device with position-adjustable virtual wall. (**ii**) Histograms of bacteria and particle counts in distribution of calculated diameter derived from impedance signals. Reproduced from [62] with the permission from Royal Society of Chemistry. (**B**) Scatter plots of impedance signal amplitude and phase changes at 2 MHz for *E. coli* and 2-μm beads. Reproduced from [135] with the permission from Elsevier. (**C**) Histograms of cell counts in distribution of impedance signal phase at 8 MHz for *E. coli*, MSSA and *E. coli* + MSSA + beads. Reproduced from [53] with the permission from MDPI. (**D**) Scatter plots of impedance signal amplitude and phase at low (366 kHz) and high frequencies (6.9 MHz) for *E. coli* with ethanol and heat treatment. Reproduced from [140] with the permission from MDPI.

Bernabini et al. demonstrated the feasibility to detect bacteria according to cell size in an IFC device [133]. This device features small cross-sectional area of the flow channel and narrow width of electrodes, since the size of bacteria is usually smaller than that of mammalian cells. Without the measurement of signal phase, *E. coli* could be identified by volume rather than membrane capacitance, and thus *E. coli* was indistinguishable from particles in similar size [133]. To solve this problem, phase metric was introduced and thus the viability and species of bacteria could be determined [134]. In order to precisely measure the diameter of different bacterium, Choi reported an IFC device with position-adjustable virtual wall [62]. The movement of virtual wall is modulated by adjusting the flow rate of sample suspension focused by low conductive sheath flow. The cross-sectional area of sample flow could be adjusted to approximate that of bacteria, making the impedance changes caused by the bacteria passage more significant (Figure 7A). Recently, Guler et al. merged the amplitude and phase information of impedance signals to achieve higher size sensitivity and detection throughput of bacteria (Figure 7B) [135]. Besides, Clausen et al. used two simple IFC devices with coplanar and facing electrodes, respectively, to detect different types of bacteria [53]. These IFC devices could be used to accurately measure any change in bacteria concentration and distinguish methicillin-sensitive *Staphylococcus aureus* (MSSA) from *E. coli* according to the impedance phase signal at 8 MHz (Figure 7C). Moreover, Bertelsen et al. detected and characterized *E. coli* inactivated by ethanol, heat and autoclaving, respectively [140]. The population of ethanol-treated bacteria showed a similar amplitude to 1.5-μm polystyrene beads, which was consistent with the hypothesis of membrane disruption. Supported by the experimental data, ethanol treatment caused membrane disruption while heat process did no obvious harm to cell membrane. In detail, the loss of membrane integrity corresponded to changes in impedance signal amplitude and phase (Figure 7D). Notably, Spencer et al. developed a method to optimize the prescription of antibiotic by an impedance-based fast antimicrobial susceptibility test (iFAST) [141]. By applying microfluidic impedance cytometry with differential electrode configuration, the phenotype response (electrical opacity and electrical diameter) of *Klebsiella pneumoniae* (*K. pneumoniae*) to specific antibiotic was accurately analyzed.

**Figure 8:**
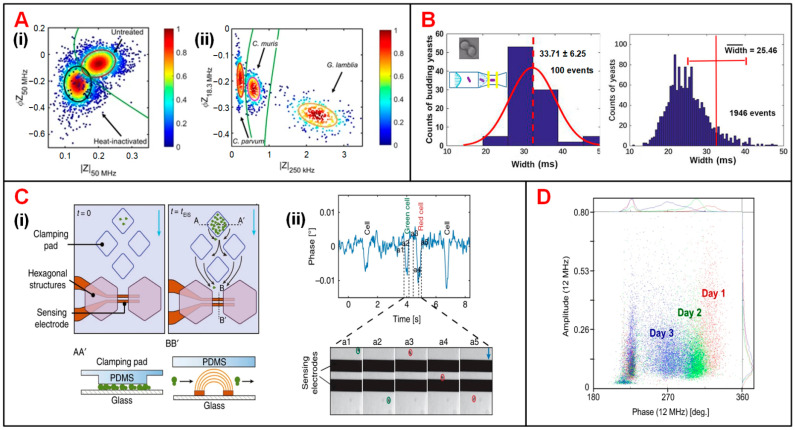
Microbial analysis using IFC devices. (**A**) (**i**) Scatter plot of phase (*Φ*Z_50MHz_) vs. amplitude (|Z_50MHz_|) for heat-inactivated and untreated *C. parvum*. (**ii**) Scatter plot of phase (*Φ*Z_18.3MHz_) vs. amplitude (|Z_250kHz_|) for *C. parvum*, *C. muris* and *G. lamblia*. Reproduced from [136] with the permission from Nature. (**B**) Histograms of particle counts in distribution of signal pulse width measured at 1 MHz. Width refers to the time that yeast cells take to pass through the sensing electrodes. Reproduced from [139] with the permission from American Chemical Society. (**C**) (**i**) Schematics of an IFC device used for long-term budding yeast culturing and growth-rate measurement. (**ii**) Signal phase changes corresponding to five events that yeast cells passing through the impedance sensing electrodes. Reproduced from [137] with the permission from Nature. (**D**) Scatter plot of impedance signal amplitude vs. phase at 12 MHz for yeast population during three-day brewing process. Reproduced from [138] with the permission from Springer.

Besides, accurate recognition and viability analysis of protozoan pathogens have advanced in parasitic diseases diagnosis of human and livestock [149]. To this end, Mcgrath et al. detected single protozoan oocysts utilizing a continuous IFC system [136]. The heat treatment performed on *Cryptosporidium parvum* (*C. parvum*) lowered the impedance signal amplitude and phase especially at high frequency representing the internal properties of the oocyst (50 MHz) (Figure 8(Ai)). The difference of viable and inactive populations can be enhanced by increasing the conductivity of medium suspension. In addition, according to the amplitude at 250 kHz and phase at 18.3 MHz, the major human-pathogenic species (*C. parvum*, *Cryptosporidium muris* (*C. muris*) and *Giardia Lamblia* (*G. lamblia*)) were discriminated from other parasite species that posed little or no risk to human health (Figure 8(Aii)).

Yeast cells, easily accessible and culturable, have been widely used as an important model organism to study cell growth and division in cell cycle progression [94]. Xie et al. optimized an IFC device with a constriction channel to detect the size of single budding yeast (*S. cerevisiae*) cells and calculate the late-budding rates of populations [139]. As shown in Figure 8B, due to the impact of velocity gradient near the constriction channel, rod particles are aligned with the electric field lines, so that the length of rod particles could be assessed by pulse width of impedance amplitude at 1 MHz. In this way, rod and spherical particles could be clearly discriminated. Moreover, late-budding yeast, namely mother cell with a daughter cell that is nearly mature, can be viewed as a rod-shaped cell, while other yeast is approximately oval. Using the same principle, the shape of target cells as well as the budding stages can be obtained. In another work, Chawla et al. developed a microfluidic platform allowing for long-term culturing and independent monitoring of growth rate of budding yeast (Figure 8(Ci)) [137]. In this device, multiple cell populations were anchored to pads and their daughter cells were then washed away, flowing through the impedance sensing unit. By analyzing the impedance signal phase at 1.5 MHz, passages of cells through electrodes were recorded. Then by counting the flowing cells in unit time, the growth rate of cell population can be calculated. As shown in Figure 8(Cii), the phase fluctuated drastically as the cell passes through the electrodes, and each phase pulse corresponds to a single cell flowing through the sensing area. This device enabled culturing and monitoring of various groups of budding yeast simultaneously. At the same time, cell populations can be exposed to different medium solution and their growth rates can be calculated indirectly from impedance signal phase. In addition, Opitz et al. focused on monitoring and analyzing of yeast population under different culturing conditions [138]. In their study, impedance signals at 12 MHz were analyzed to characterize cell viability in a three-day brewing process. On the first day, the high phase indicated that the cell population had high viability and they began to breed by large numbers (Figure 8D). By the end of the third day, the cell population showed lower viability. The cell loss could be ascribed to the depletion of oxygen and the accumulated ethanol.

### 4.2. EIS to Detect Suspended or Adherent Single Cells

Different from IFC devices that are commonly used for cell recognition and screening with high throughput, EIS sensing devices are capable of extracting broadband impedance information and tracking dynamic variations of single cells. Recent EIS sensing devices applied in single-cell analysis are summarized in Table 2. These devices are classified into two categories: one is to determine the optimal frequency at which the impedance of different cell lines or cell states is most sensitive [82,150] and the other is to continuously monitor the dynamic cell process or cell behavior and phenotypic changes [83,85,91,92,94,95,148,151,152,153].

**Figure 9:**
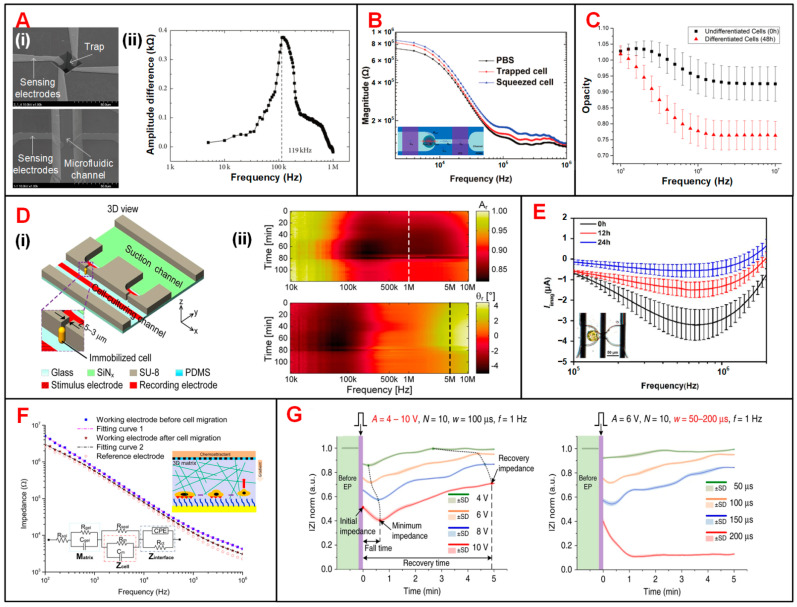
Cell-based assay using EIS sensing devices. (**A**) (**i**) SEM images of the two devices used to detect cancerous urothelial cells. Left one is an EIS sensing device with a negative pressure trap used to investigate the optimal frequency. Right one is an IFC device to perform high-throughput electrical impedance measurement of normal and cancerous urothelial cells. (**ii**) Measurement of the amplitude difference between normal and cancerous urothelial cells in the frequency range of 5 kHz to 1 MHz. Reproduced from [150] with the permission from Hindawi. (**B**) Schematics of a EIS sensing device to measure the amplitude and phase signal of MCF-7 cells under three typical conditions: PBS solution without cells, cell trapped and cell squeezed. Reproduced from [82] with the permission from Springer. (**C**) Using a EIS sensing device with microfluidic traps to distinguish the undifferentiated and differentiated cells by measuring the impedance over the frequency range from 100 kHz to 10 MHz. Reproduced from [95] with the permission from Elsevier. (**D**) (**i**) Schematics of an EIS-integrated single-cell culturing device for immobilization and impedance recording of *Schizosaccharomyces pombe* (*S. pombe*) cells. (**ii**) Recorded EIS amplitude and phase signals over the frequency range from 10 kHz to 10 MHz showing the growth and division of single *S. pombe* cells. Reproduced from [94] with the permission from Nature. (**E**) Imaginary part of current response for *Arabidopsis* mesophyll cells at different status (0 h, 12 h and 24 h after incubation, respectively). Reproduced from [148] with the permission from Elsevier. (**F**) The Bode impedance spectra measured on working electrode before and after cell migration, as well as on reference electrode without cells over the frequency range from 100 Hz to 1 MHz. Reproduced from [85] with the permission from American Chemical Society. (**G**) Recording of |Z|_norm_ for HeLa cells in the recovery process under different conditions of electroporation. *A*, *N*, *w* and *f* stand for pulse amplitude, number, width and frequency, respectively. Reproduced from [153] with the permission from Nature.

EIS sensing technology has been used to investigate the optimal frequency at which the characteristic parameters extracted from EIS signals are most prominent in measuring specific dielectric properties of cells [82,150]. Park et al. proposed two types of devices to distinguish cancerous from normal human urothelial cell lines (Figure 9(Ai)) [150]. In one device, single cells were captured at 3D traps by applying negative pressure underneath. Then, the impedance of immobilized single cells was individually measured at frequencies from 5 kHz to 1 MHz. According to the EIS signals in Figure 9(Aii) plot, 119 kHz was supposed to be the optimal frequency, at which the impedance of two types of cells had the greatest divergence. The real-time impedance of the cell lines was measured at 119 kHz in the other device (an IFC device) to identify cancerous cells. These two devices potentially provide a supplementary platform to detect urothelial cancer of the bladder (UCB). In another study, Tang et al. developed a portable single-cell analytical system combining hydrodynamic traps and EIS measurement to accurately detect the sizes of MCF-7 cells [82]. Under the hydrodynamic forces, MCF-7 cells could be initially captured at the entrance of the narrow channel and then squeezed into it. Impedance signals were collected from three groups, among which one is the control group of PBS solution without cells, another one is the trapped cells in suspension, and the third one is the squeezed cells (Figure 9B). According to the sweep-frequency measurement of EIS, the frequency was optimized to 500 kHz, at which, cellular trapping-releasing-squeezing manipulation and cell size could be detected more accurately.

EIS sensing technology has been used to monitor cell behavior and phenotypic changes, including differentiation of stem cells [92,148,154], cell growth and division [83,94,155], formation of cell wall [95], migration of tumor cells [85,152] and recovery process after electroporation [153].

In order to characterize the differentiation process of stem cells, Zhou et al. analyzed the impedance data from mouse embryonic stem cells (mESCs) at different time points in a cell differentiation cycle [95]. In this study, impedance opacity (|Z_1MHz_|/|Z_50kHz_|) was increasing during the 48-h cell differentiation process, and was significant at above 1 MHz (Figure 9C). Based on this finding, they observed the metastable transition state, from which stem cells could either differentiate irreversibly or return to pre-differentiation state at 24 h. Zhang et al. proposed a multifunctional microfluidic chip, which featured DEP trapping, electrical stimulation and real-time impedance monitoring of single cells [92,153,154]. They recorded the real-time impedance changes of two groups of MSCs with (OM group) or without electrical stimulation (OM + ES group) [92]. The results showed that electrical stimulation could accelerate the response to drug and advance the differentiation of MSCs. Besides, this device provided additional phenotypic indicators that were not available in cell traction force sensor and contributed to multimodal characterization of long-term physiological variations in the cell differentiation process [154].

Ghenim et al. were the first to monitor the impedance variation in the mitosis of a single mammalian cell [155]. Zhu et al. presented a microfluidic cell-culturing chip to trap, cultivate and selectively release individual yeast cells [156]. Then, this device was used to monitor the cell dynamics in a cell cycle of yeast cells (Figure 9(Di)) [83,94]. As an example, electrodes originally used to generate DEP forces were used to measure the electrical impedance spectrum of rod-shaped *S. pombe* cells, which were immobilized in an upright position at the traps [94]. Cell growth, nuclear division and cytokinesis in a cell cycle were sensitively characterized by EIS signal amplitude at 1 MHz and phase at 5 MHz (Figure 9(Dii)).

Chen et al. investigated the formation process of primary cell wall of *Arabidopsis* mesophyll cells [148]. As discussed in Section 4.1.4, the formation of the cell wall reduced the capacitance of entire plant cell and thus led to an increase in the imaginary part of impedance signal [60]. In support of this hypothesis, they measured the differential current response of *Arabidopsis* mesophyll cells at three status of cell wall formation (Figure 9E).

Cell migration, which serves as the initiation of cancer metastasis, could be recorded by ECIS technology [157]. Primiceri et al. demonstrated that cell migration could be monitored and automatically analyzed by a EIS biochip [152]. Nguyen et al. proposed a microfluidic chip with ECIS for monitoring the migration of single cancer cells in 3D matrixes (Figure 3C) [85]. In this study, the impedance measurements were performed with a voltage of 10 mV over the frequency range from 100 Hz to 1 MHz and showed the significant decrease of EIS amplitude after cell migration (Figure 9F). The real-time EIS recording was carried out at 4 kHz and demonstrated that MCF-7 cells were less metastatic than MDA-MB-231 cells. Zhang et al. monitored the recovery processes of HeLa cells after electroporation by using impedance measurement (Figure 9G) [153]. HeLa cells were trapped and electroporated with different working modes of center electrodes. Within 5 min after electroporation, normalized amplitude curves were slowly rising corresponded to the reversible EP processes, while those stabilizing at the minimum values indicated the irreversible EP and cell death.

## 5. Conclusions

Electrical impedance sensing technology, as a rapid and non-invasive method to probe cellular biophysical information, has become appealing in single-cell study. The basic theories and modeling methods of single-cell impedance sensing have been re-viewed herein and recent advances in this field have been highlighted with respect to the device design and applications.

Generally, the way to implement electrical impedance measurement in a microfluidic device is categorized into IFC and EIS sensing. IFC features measuring impedance of single frequencies for large number of cells, while EIS sensing is capable of re-al-time monitoring of a few cells over a wide frequency range. A variety of optimal electrode layouts, fluidic channel configurations, hydrodynamic focusing systems have been proposed to improve the sensitivity and consistency of IFC in the measurement of cellular electrical parameters. With various trapping methods, suspended single cells could be stably immobilized for *in-situ* EIS sensing. ECIS can recognize cellular behavior sensitively in response to defined stimulus. Besides, individually addressable MEAs have been incorporated into EIS sensing devices in order to overcome the limitation of throughput. Impedance converters and LIAs, as the basic electronic components to measure impedance, have been further integrated and miniaturized from instruments to portable platforms. As an alternative, CMOS-based impedance sensing devices have been developed to increase the integration level of impedance sensing system.

Since IFC devices have the merits of rapid measurement and high throughput, they have been widely used in the identification and classification of various species of single cells and determination of cell viability. The impedance signals at different frequencies reveal dielectric characteristics of different cellular structures. Hence, a combination of multiple impedance parameters, such as amplitude at high frequency and low frequency or amplitude and phase at the same frequency, has been commonly used to identify single cells with various phenotypes, in different life stages or under multiple external conditions. Applications of the IFC devices in plant cell analysis, especially pollen screening, have been proposed and promoted, and the strategy of combining mechanical characterization and impedance measurement has been also developed. In addition, machine learning has been used in impedance data analysis to improve the performance of IFC devices.

EIS sensing has been used to choose the most sensitive frequency for subsequent high-speed analysis or long-term monitoring of cell behavior and phenotypes. The various cellular physiological processes, including adhesion, growth, division, differentiation, proliferation and cellular structure formation, have been characterized by the measured electrical impedance spectra.

From this review, insights into challenges and prospects of electrical impedance sensing technology for single-cell analysis could be provided as follows. Different cell subpopulations are hard to be accurately classified based on impedance information at specific frequencies. To this end, multi-frequency impedance signals and the combination of multiple biophysical parameters could be used to enrich characteristic information of different cells, and thus could favor the improvement of phenotyping resolution. Besides, machine learning algorithm, such as SVM and neural networks, in data analysis could help to correspond broadband impedance signals to cell phenotypic characteristics or single-cell physiological processes. On the other hand, the high cost of chip fabrication and benchtop instruments hinders the popularization of single-cell impedance sensing technology. It can be supposed that single-cell impedance measurement devices could soon appear in the clinical laboratory in a more user-friendly format, with the help of development and promotion of commercial equipment and portable platforms. By then, a quantum leap will appear in the fields of rapid diagnosis, smart healthcare and personalized medicine.

## Figures and Tables

**Table 1:** Applications of IFC for single-cell analysis.

Category	First Author (Year)	Electrode and Fluidic Layouts	Frequency	Target Cells	Application	Ref.
Blood cells	Holmes (2010)	2 coplanar electrode pairs	503 kHz and 10 MHz	CD4 T-cells	Cell counting	[112]
	Du (2013)	1 coplanar electrode pair	2 MHz	Red blood cells	Detection of malaria-infected cells	[113]
	Hassan (2016)	2 coplanar electrode pairs	303 kHz and 1.7 MHz	CD4 and CD8 T-cells	Cell counting	[111]
	Liu (2018)	2 coplanar electrode pairs	156 kHz, 500 kHz and 3 MHz	Red blood cells	Detection of sickle cells	[110]
	Honrdo (2018)	2 facing electrode pairs, fluorescence detection	2–8 MHz	Red blood cells	Detection of malaria-infected cells	[114]
Tumor cells	Choi (2013)	Two polyelectrolyte gel electrodes	DC	OVCAR-3 cells	Cell recognition	[115]
	Spencer (2014)	2 facing electrode pairs	0.5 MHz and 2 MHz	MCF-7 cells	Cell recognition	[52]
	Han (2015)	2 facing electrode pairs	500 kHz and 10 MHz	DLD-1 cells	Cell recognition	[116]
	Zhao (2016)	μCPC with constriction channel	1 kHz and 100 kHz	A549 and H1299 cells	Cell screening	[117]
	Desai (2019)	2 coplanar electrode pairs, sheath flow focusing	250 kHz	Thyroid, breast, lung, and ovarian cancer cells	Cell recognition	[118]
	Ren (2019)	1 coplanar electrode pair, 2 constriction channels	1 kHz, 10 kHz, 100 kHz, and 1 MHz	MDA-MB-231 cells	Cell recognition	[119]
	McGrath (2020)	5 facing electrode pairs	500 kHz–50 MHz	Six types of pancreatic ductal adenocarcinoma cell	Cell screening	[120]
	Ostermann (2020)	2 facing electrode pairs	6 MHz	U937 cells	Viability assay	[121]
	Zhang (2020)	1 coplanar electrode pair, asymmetrical constriction channel	100 kHz and 250 kHz	A549 and Hep G2 cells	Cell screening	[43]
Stem cells	Song (2016)	C-shaped arranged coplanar electrodes	500 kHz and 3 MHz	Mesenchymal stem cells	Monitoring differentiation process	[125]
	Xavier (2017)	2 facing electrode pairs, fluorescence detection	500 Hz and 2MHz	Skeletal stem cells	Monitoring differentiation process	[127]
Plant cells	Heidmann (2016)	2 facing electrode pairs	500 Hz and 12 MHz	Tobacco pollen	Viability assay	[128]
	Heidmann (2017)	2 facing electrode pairs	500 kHz, 3 MHz and 12 MHz	Tomato, pepper, potato and wind pollinators pollen	Viability assay	[129]
	Impe (2019)	2 facing electrode pairs	1 MHz	Wheat pollen	Viability assay	[130]
	Ascari (2020)	2 facing electrode pairs	2 MHz and 8 MHz	Hazelnut pollen	Viability assay	[131]
	Canonge (2020)	2 facing electrode pairs	500 kHz and 12 MHz	Wheat microspore	Monitoring androgenesis process	[132]
	Han (2020)	2 coplanar electrode pairs, constriction channel	500 kHz and 5 MHz	Herbaceous *Arabidopsis* *thaliana* and woody *Populus trichocarpa*	Cell screening	[60]
Microbes	Choi (2014)	2 polyelectrolytic gel electrodes, sheath focusing	DC	*F. tularensis* and *E. coli*	Cell recognition	[62]
	Mcgrath (2017)	2 facing electrode pairs	250 kHz, 18.3 MHz and 50 MHz	*C. parvum*	Viability assay	[136]
	Guler (2018)	1 coplanar electrode pairs	2 MHz	*E. coli*	Cell recognition	[135]
	Clausen (2018)	2 coplanar electrode pairs 2 facing electrode pairs	200 kHz and 7 MHz	*E. coli*	Cell recognition	[53]
	Chawla (2018)	1 coplanar electrode pairs	1.12 MHz and 1.5 MHz	*S. cerevisiae* cells	Monitoring cell growth rate	[137]
	Xie (2019)	1 coplanar electrode pairs	1 MHz	*S. cerevisiae* cells	Reproductive performance assessment	[139]
	Opitz (2019)	2 facing electrode pairs	0.5 MHz, 10 MHz and 12 MHz	*S. cerevisiae* cells	Viability assay	[138]
	Bertelsen (2020)	2 facing electrode pairs	366 kHz and 6.9 MHz	*E. coli*	Determination of the viability of *E. coli*	[140]
	Spencer (2020)	4 facing electrode pairs	5 MHz and 40 MHz	*K. pneumoniae*	Antimicrobial susceptibility tests	[141]

**Table 2:** Applications of EIS measurement for single cells. OT: Observation time. Throughput: Maximum number of single cells that can be simultaneously measured.

First Author (Year)	Techniques	Frequency Range	Throughput	OT	Target Cells	Application	Ref.
Primiceri (2011)	ECIS	1 Hz to 1 MHz	/	4 h	Hepatocellular carcinoma cells	Monitoring cell migration	[152]
Hong (2012)	DEP traps	20 kHz to 101 kHz	/	/	A549, MDA-MB-231, MCF-7, and HeLa cells	Electrical characteristics analysis of cancer cells	[151]
Nguyen (2013)	Hydrodynamic traps and ECIS	100 Hz to 1 MHz	16	/	MDA-MB-231 and MCF-7 cells	Monitoring cell capture, adhesion, and spreading process	[85]
Zhu (2014)	Negative pressure traps	10 kHz to 10 MHz	10	42 min	*S. cerevisiae* cells	Monitoring bud growth and cell motion	[83]
Zhu (2015)	Negative pressure traps	10 kHz to 10 MHz	10	120 min	*S. pombe* cells	Cell cycle determination	[94]
Zhou (2016)	Hydrodynamic traps	100 Hz to 20 MHz	10	48 h	Mouse embryonic stem cells	Monitoring the differentiation process	[95]
Park (2016)	Negative pressure traps	5 kHz to 1 MHz	5	/	Cancerous human urothelial cells (TCCSUP)	Cell recognition	[150]
Tsai (2016)	Hydrodynamic traps	10 kHz to 100 kHz	3	24 h	HeLa cells	Monitoring electrical characteristics	[91]
Tang (2017)	Hydrodynamic traps	1.953 kHz to 1 MHz	10	/	MCF-7 cells	Monitoring the capture process and cell screening	[82]
Chen (2020)	Hydrodynamic traps	100 kHz to 2 MHz	/	24 h	*Arabidopsis* mesophyll cells	Monitoring the regeneration process of primary cell wall	[148]
Zhang (2020)	DEP traps and ECIS	100 kHz	32	5 min	HeLa, MCF-7, and 293T cells	Monitoring the recovery process after electroporation	[153]
Zhang (2020)	DEP traps and ECIS	100 kHz	32	21 days	Mesenchymal stem cells	Monitoring differentiation process	[92]

## Data Availability

Not applicable.
